# Hydroxychloroquine prophylaxis and treatment is ineffective in macaque and hamster SARS-CoV-2 disease models

**DOI:** 10.1172/jci.insight.143174

**Published:** 2020-12-03

**Authors:** Kyle Rosenke, Michael A. Jarvis, Friederike Feldmann, Benjamin Schwarz, Atsushi Okumura, Jamie Lovaglio, Greg Saturday, Patrick W. Hanley, Kimberly Meade-White, Brandi N. Williamson, Frederick Hansen, Lizette Perez-Perez, Shanna Leventhal, Tsing-Lee Tang-Huau, Julie Callison, Elaine Haddock, Kaitlin A. Stromberg, Dana Scott, Graham Sewell, Catharine M. Bosio, David Hawman, Emmie de Wit, Heinz Feldmann

**Affiliations:** 1 Laboratory of Virology, National Institute of Allergy and Infectious Diseases, NIH, Hamilton, Montana, USA.; 2 University of Plymouth, Plymouth, Devon, United Kingdom; The Vaccine Group Ltd, Plymouth, Devon, United Kingdom.; 3 Rocky Mountain Veterinary Branch and; 4 Laboratory of Bacteriology, Division of Intramural Research, National Institute of Allergy and Infectious Diseases, NIH, Hamilton, Montana, USA.; 5 Leicester School of Pharmacy, De Montfort University, Leicester, United Kingdom.

**Keywords:** COVID-19, Therapeutics, Cytokines, Drug screens, Molecular biology

## Abstract

We remain largely without effective prophylactic/therapeutic interventions for COVID-19. Although many human COVID-19 clinical trials are ongoing, there remains a deficiency of supportive preclinical drug efficacy studies to help guide decisions. Here we assessed the prophylactic/therapeutic efficacy of hydroxychloroquine (HCQ), a drug of interest for COVID-19 management, in 2 animal disease models. The standard human malaria HCQ prophylaxis (6.5 mg/kg given weekly) and treatment (6.5 mg/kg given daily) did not significantly benefit clinical outcome, nor did it reduce SARS-CoV-2 replication/shedding in the upper and lower respiratory tract in the rhesus macaque disease model. Similarly, when used for prophylaxis or treatment, neither the standard human malaria dose (6.5 mg/kg) nor a high dose (50 mg/kg) of HCQ had any beneficial effect on clinical disease or SARS-CoV-2 kinetics (replication/shedding) in the Syrian hamster disease model. Results from these 2 preclinical animal models may prove helpful in guiding clinical use of HCQ for prophylaxis/treatment of COVID-19.

## Abstract 2

The use of hydroxychloroquine was assessed as a possible therapeutic in two COVID-19 animal models and was found to be ineffective.

## Graphical abstract

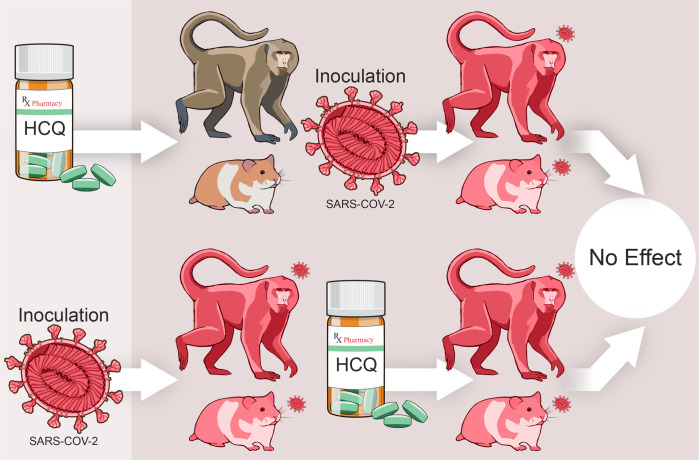

## Introduction

Severe acute respiratory syndrome coronavirus 2 (SARS-CoV-2) is the causative agent of coronavirus disease 2019 (COVID-19) (1). SARS-CoV-2 infections were initially reported in China near the beginning of December 2019 (2). After early spread through Asia, and subsequently to European, American, and African countries, the virus is responsible for the third pandemic of the 21st century. With currently (late July, 2020) over 48 million confirmed cases and greater than 1.2 million deaths worldwide, health systems are stretched beyond their limits with largely no proven treatment or prophylaxis available to reduce the burden (3). Public health measures combined with increasingly severe restrictions on public life have been implemented in many countries to stop SARS-CoV-2 transmission. The goal of public health strategies is still to flatten the epidemiologic SARS-CoV-2/COVID-19 curve to ease the burden on health care systems challenged by the highly intensive care required for a significant proportion of COVID-19 cases. Over 1000 clinical trials are currently open or being established in different countries testing drugs such as lopinavir/ritonavir, dexamethasone, hydroxychloroquine (HCQ), and inhaled IFN-β–1a (4). Yet, many of these treatments have not been empirically tested in relevant SARS-CoV-2 animal disease models to determine preclinical efficacy, which would possibly provide valuable insight into prioritization of drugs to move forward in humans.

At the time this work was started, the FDA had given emergency approval for the use of chloroquine and HCQ in patients with COVID-19 (5). In vitro data on the inhibitory effect of chloroquine and HCQ on SARS-CoV-2 replication had been published (6–8) and HCQ alone or in combination with the macrolide antibiotic azithromycin had been used in early clinical trials to treat COVID-19 cases with varying effect (9–11). Despite ongoing clinical trials, preclinical efficacy data on the effect of HCQ in SARS-CoV-2 animal disease models were lacking. Herein, we assessed the efficacy of HCQ prophylaxis and treatment in 2 established animal disease models, the Syrian hamster and rhesus macaque (12, 13).

## Results

First, we confirmed the in vitro inhibitory effect of HCQ on SARS-CoV-2 replication in Vero E6 cells. Cells were pretreated with differing drug concentrations and the effect on viral RNA load in tissue culture supernatant was determined 72 hours after infection by quantitative reverse transcriptase PCR (qRT-PCR) (Supplemental Figure 1; supplemental material available online with this article; https://doi.org/10.1172/jci.insight.143174DS1). The half-maximal effective concentration (EC_50_) value for HCQ in our studies was 164.7 nM. Published EC_50_ values for inhibitors of SARS-CoV-2 have been shown to vary considerably (~2-logs) even within the same cell type (14), which is presumably due to differences in methods of analyses used in the different studies. Although the value of 164.7 nM is lower than seen in other studies, it is consistent with low/submicromolar levels (0.7 to 17 uM) previously reported for the established in vitro inhibitory effect of HCQ on SARS-CoV-2 replication in Vero E6 cells (7, 8, 14).

Having confirmed in vitro efficacy, next, we assessed HCQ efficacy in the rhesus macaque, the only animal model at that time displaying mild-to-moderate COVID-like disease upon SARS-CoV-2 infection (13). We investigated the effect of HCQ when administered either prophylactically or as a treatment after infection. A wide variety of different HCQ-dosing protocols are used in both preexposure and postexposure prophylaxis studies as well as for treatment of patients with COVID-19 (4, 15, 16). Our HCQ prophylaxis study design was based on the approved Plaquenil regimen, which comprises a single weekly dose (6.5 mg/kg) to ensure adequate tissue loading (17, 18). Similarly, for therapeutic treatment, the recommended treatment for malaria of 6.5 mg/kg was followed, except that the initial 13 mg/kg loading dose was divided over 2 boluses 6 hours apart (17, 18). This treatment regimen has demonstrated established efficacy against malaria infection with minimal toxicity.

**Figure 1:**
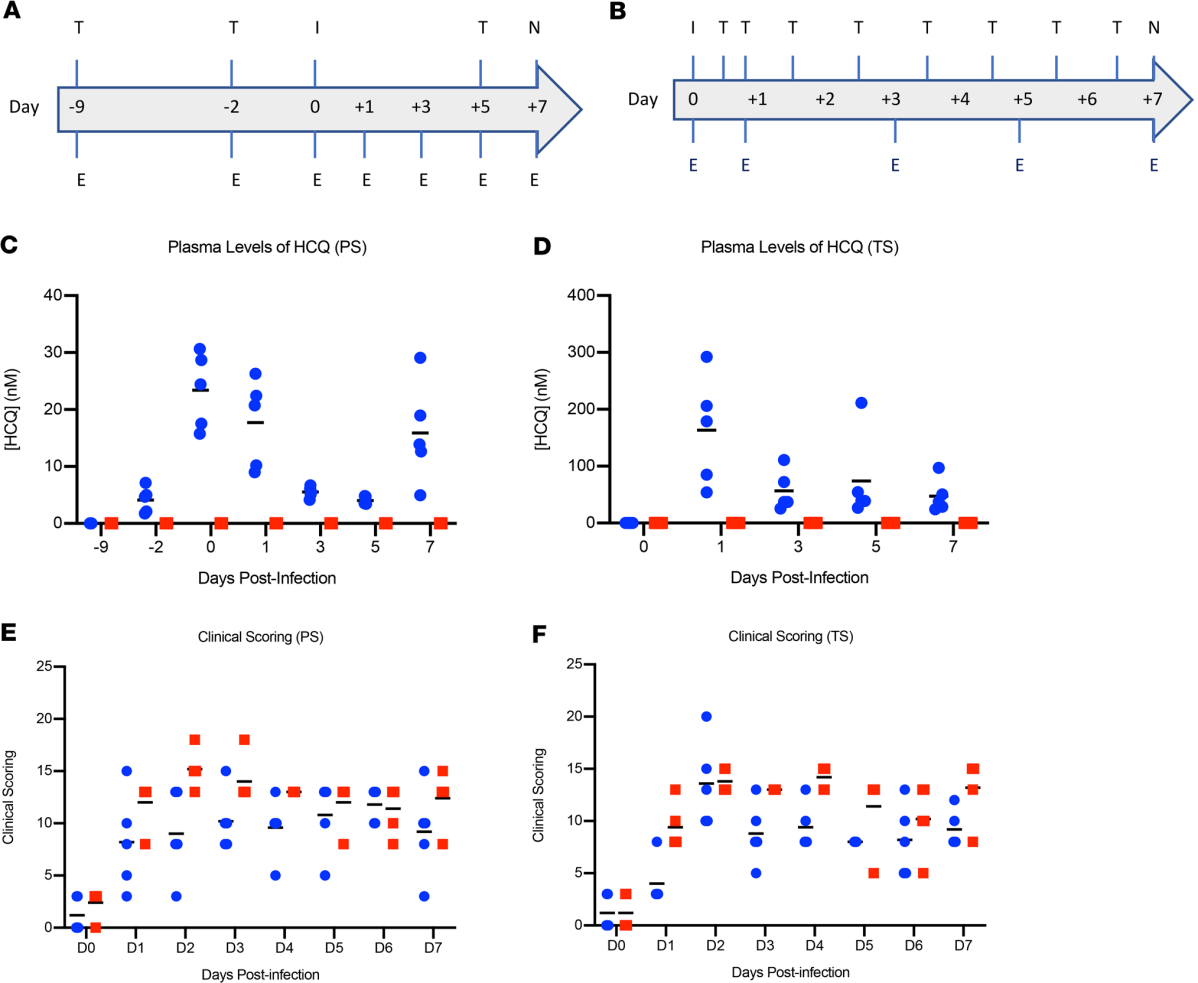
Rhesus macaque model — design, drug concentrations, and clinical scoring. Macaques were infected with SARS-CoV-2 by the combined intratracheal, intranasal, oral, and ocular routes. Animals were treated by oral gavage with either vehicle (PBS) or HCQ (6.5 mg/kg in PBS). Administration was either 1 time per week for the prophylaxis arm or starting 12 hours after infection followed by treatment 18, 36, 60, 84, 108, 132, and 156 hours after infection for the treatment arm. Animals were scored for clinical disease twice daily and examinations were performed as indicated. (**A** and **B**) Study design. The schematic depicts SARS-CoV-2 infection (I), HCQ or vehicle treatment (T), examinations (E), and necropsy (N). (**C** and **D**) Plasma levels of HCQ. HCQ levels were determined in both the prophylaxis and treatment study arms. Measurements reflect predose levels of HCQ at each time point (limit of quantification = 1.5 nM). (**E** and **F**) Clinical scores. Clinical scoring was performed twice daily by observation of nonanesthetized animals. The morning score is graphed here. Red squares indicate vehicle-treated animals and blue circles indicate HCQ-treated animals. SARS-CoV-2, severe acute respiratory syndrome coronavirus 2; HCQ, hydroxychloroquine; PS, prophylaxis; TS, treatment.

For the prophylactic arm, 10 healthy rhesus macaques were randomly divided into vehicle control and HCQ prophylaxis groups (*n* = 5 per group). Animals were treated by oral gavage with either vehicle (PBS) or HCQ (6.5 mg/kg in PBS) 3 times 1 week apart (day –9, day –2, and day 5) (Figure 1A). To test the efficacy of HCQ as a treatment, a separate group of 10 healthy rhesus macaques were randomly divided into vehicle control and HCQ treatment groups (*n* = 5 per group). Animals were treated by oral gavage with either vehicle (PBS) or HCQ (6.5 mg/kg in PBS) starting 12 hours after infection followed by treatment 18, 36, 60, 84, 108, 132, and 156 hours after infection (Figure 1B). Animals in all groups were infected on day 0 with a total dose of 2.8 × 10^6^ median tissue culture infectious doses (TCID_50_) of SARS-CoV-2 by a combination of 4 routes (intratracheal, oral, intranasal, and ocular) as previously established (13). Animals were monitored at least twice daily using an established scoring sheet designed to assess clinical signs of disease (13, 19). Multiple physical examinations were performed on different days preinoculation and after inoculation including a clinical evaluation, radiographs, blood collection, and swabs (oral and nasal). Bronchoalveolar lavage (BAL) was performed on days 3, 5, and 7 (postmortem) (Figure 1, A and B). The endpoint for both studies was day 7 after infection, at which time all animals were euthanized and necropsied. This time point represents the best compromise between assessment of clinical progression and viral replication kinetics in tissues.

To assess the pharmacokinetics of HCQ in the animals, HCQ and its secondary metabolites were measured immediately before redosing, thus reflecting the minimum plasma concentration over the experiment. HCQ was detected in plasma samples in all prophylactically or therapeutically treated animals, with concentration ranging from 2 to 31 nM and 24 to 292 nM, respectively (Figure 1, C and D). HCQ was also detected in lung tissue at time of necropsy in all prophylactically or therapeutically treated animals, ranging from 1.5 to 12.5 nmol/g tissue and 4.1 to 34.3 nmol/g tissue, respectively. These numbers are in good agreement with the reported long half-life and large volume of distribution of HCQ (20–24). HCQ cytochrome p450 catalyzed secondary amine metabolites desethylchloroquine and desethylhydroxychloroquine and the primary amine metabolite bisdesethylchloroquine are considered to be active forms of the drug in other disease models (25). Both desethylchloroquine and desethylhydroxychloroquine were detected in intermediate concentrations, whereas trace amounts of bisdesethylchloroquine was detected in lung homogenate, suggesting persistence of active drug forms over the course of treatment (Supplemental Figure 2). The minimum plasma HCQ levels measured here are comparable with plasma levels found in human prophylactic and therapeutic ranges for malaria prevention and are consistent with the expected low plasma levels 12 to 24 hours after administration (26, 27). Based on these previously published studies, the initial maximum concentration of HCQ is expected to be 2 to 3 orders of magnitude higher than these minimum values with a rapid clearance from the bloodstream into the tissue (21). Because SARS-CoV-2 is a respiratory disease, levels of drug in lung tissue are presumably the best indicator of therapeutic potential. Volume/concentration can be difficult to estimate in tissues due to compartmentalization of the drug, resulting in a nonhomogenous distribution. On average, water content of the lung is approximately 80% by weight and this number can be used to calculate an estimated HCQ concentration in the tissue, assuming a homogenous distribution (28). Using these values, levels in the lung on day 7 were approximately 3.0 μM at a minimum. As indicated, published EC_50_ values vary considerably; however, this value of approximately 3.0 μM falls within the range of previously published (7, 8, 14) in vitro EC_50_ values for HCQ (0.7 to 17 μM).

Macaques in both the prophylactic and treatment arms of the study first displayed clinical signs of SARS-CoV-2 infection on day 1, which peaked on day 2 and animals remained mildly to moderately ill until the study endpoint on day 7 (Figure 1, E and F). Clinical signs included reduced appetite and ruffled fur followed by pale appearance and irregular increased abdominal respiration (Supplemental Table 1). Macaques did not develop fevers or coughs after SARS-CoV-2 infection and weight loss was minimal between groups. Overall, animals in the vehicle-treated groups appeared to have slightly higher clinical scores throughout; however, multiple *t* tests performed on individual days found no significant differences between groups. Hematology and serum chemistry were unremarkable for all animals in both study arms. Radiographic signs in the prophylaxis, treatment, and control groups were minimal over the study course (Supplemental Figure 3). Pulmonary infiltrates, when seen, were noted to be of a mild unstructured interstitial pattern. The pattern was rarely seen in the upper lung, being more commonly found in middle and caudal lung lobes. No differences were noted in severity or appearance of radiographic signs between HCQ prophylaxis, treatment, or control groups.

**Figure 2:**
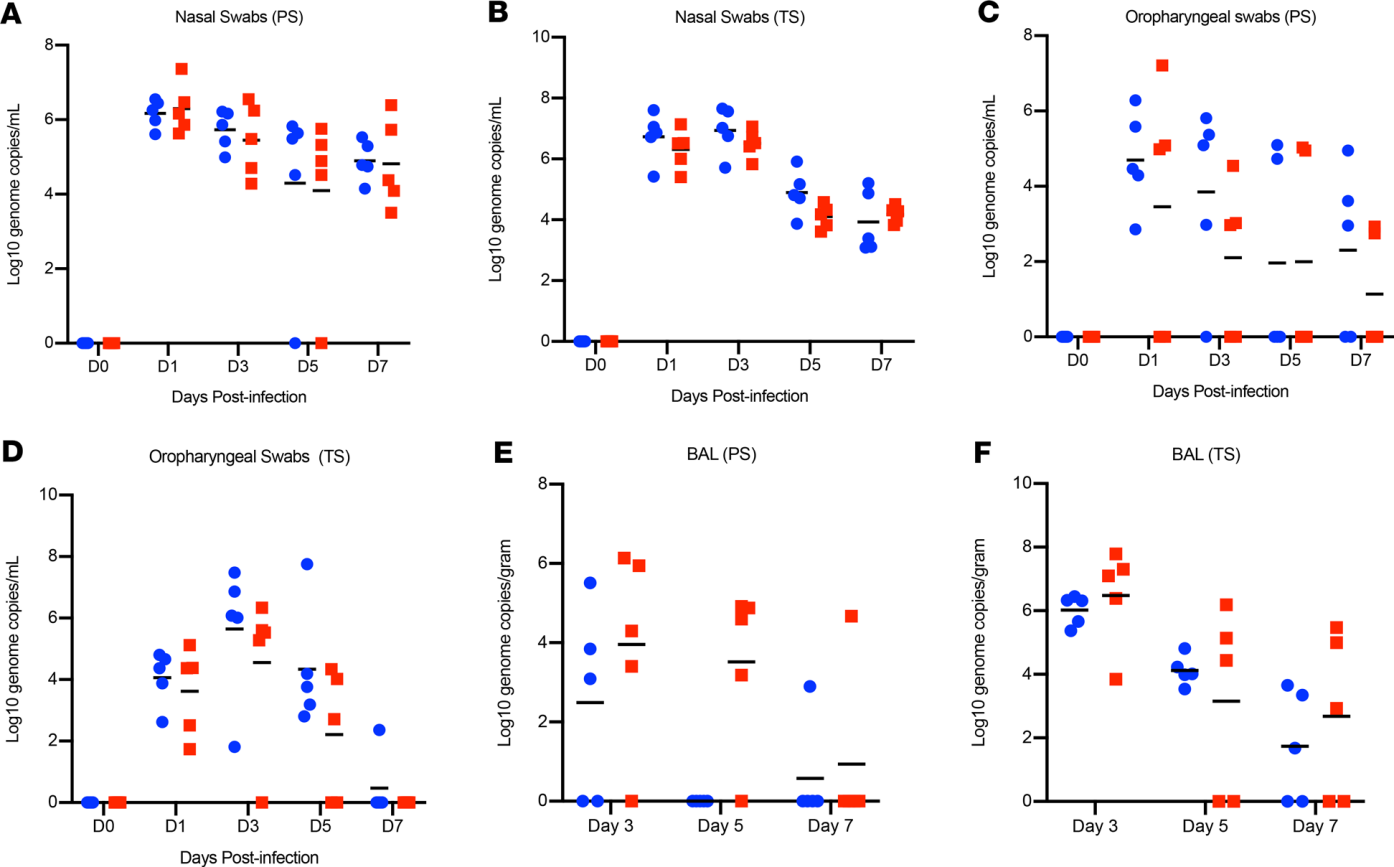
Rhesus macaque model — viral loads in lower and upper respiratory tract. Macaques were infected with SARS-CoV-2 as described in the legend of Figure 1. Swab samples (nasal and oropharyngeal) and BAL were collected at all or indicated examination time points. Viral loads were determined by qRT-PCR using the subgenomic E assay as genome copies. (**A** and **B**) Nasal swabs. (**C** and **D**) Oropharyngeal swabs. (**E** and **F**) BAL. No statistical significance was found among the groups presented in **A**–**F**. Red squares indicate vehicle-treated animals and blue circles indicate HCQ-treated animals. SARS-CoV-2, severe acute respiratory syndrome coronavirus 2; BAL, bronchoalveolar lavage; qRT-PCR, quantitative reverse transcriptase PCR; HCQ, hydroxychloroquine; PS, prophylaxis; TS, treatment.

Nasal and oropharyngeal swabs were positive for SARS-CoV-2 RNA in all animals of both studies, with the highest load on either day 1 or day 3, which then gradually decreased until the end of the study (Figure 2, A–D). Viral loads were consistently higher in nasal swabs than oropharyngeal swabs. BAL samples were collected on days 3, 5, and 7 (postmortem) and viral loads were similar to nasal and oropharyngeal swabs, with decreasing loads over time (Figure 2, E and F). Overall, there were no statistically significant differences in virus load and shedding between HCQ-administered and vehicle-administered animals in the prophylaxis and treatment regimens.

**Figure 3:**
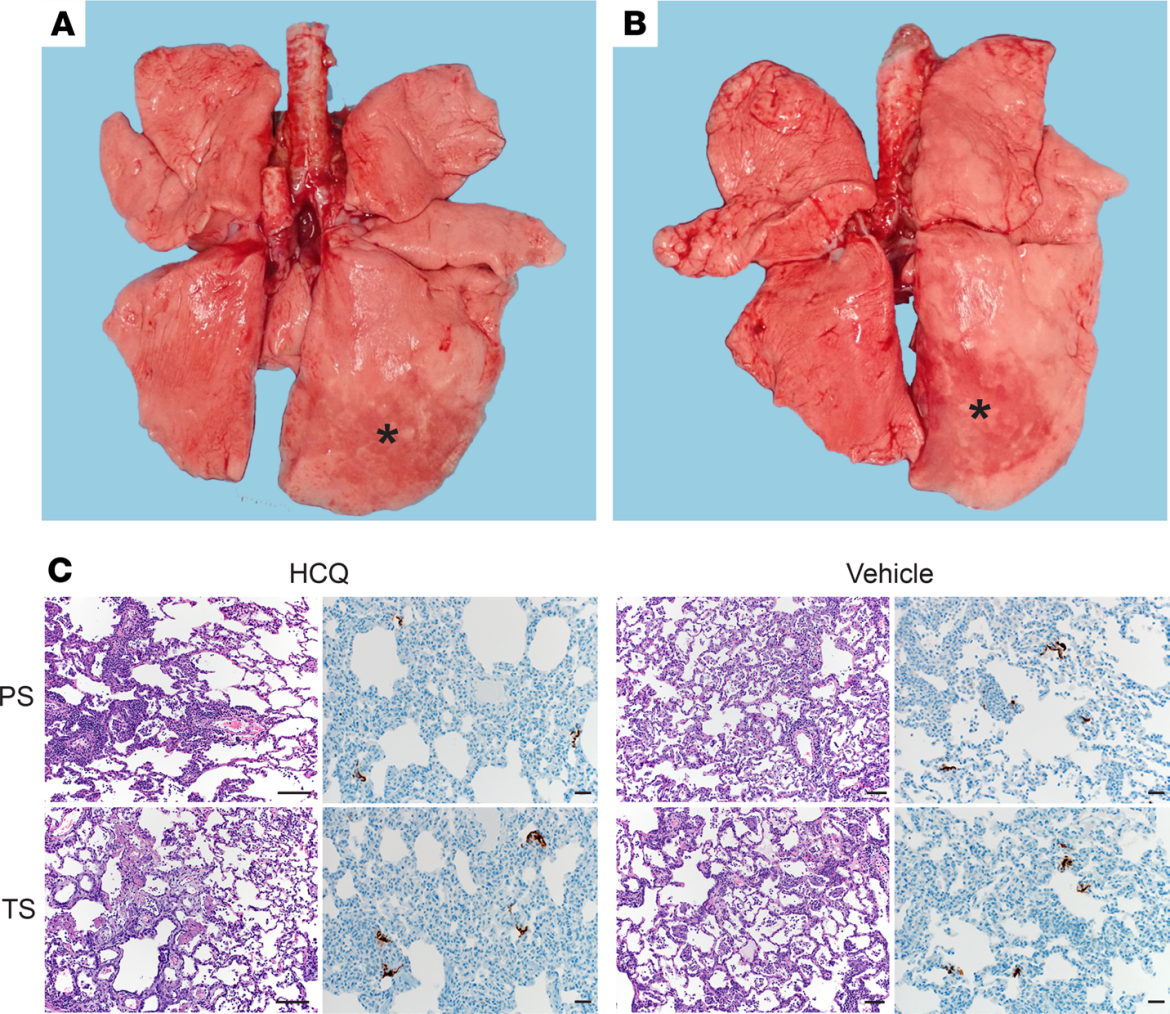
Rhesus macaque model — gross and histopathology. Macaques were infected with SARS-CoV-2 as described in the legend of Figure 1. Animals were euthanized on day 7 after infection for gross pathology and histopathology. (**A** and **B**) Gross pathology with consolidated lower left lung lobe and area of postmortem BAL in the lower right lung lobe (asterisk). (**C**) H&E (original magnification, ×100) and IHC (original magnification, ×200). H&E staining in both the hydroxychloroquine and vehicle groups revealed multifocal, minimal-to-moderate, interstitial pneumonia frequently centered on terminal bronchioles. Alveolar edema and fibrin with formation of hyaline membranes were seen only in lungs with moderate changes. Multifocal perivascular infiltrates of small numbers of lymphocytes that form perivascular cuffs. Corresponding IHC showing immunopositivity in type I and II pneumocytes. Black measurement bars represent 50 μm for H&E and 20 μm for IHC figures. SARS-CoV-2, severe acute respiratory syndrome coronavirus 2; BAL, bronchoalveolar lavage; PS, prophylaxis; TS, treatment.

At necropsy, gross pathology revealed consolidated lungs in animals of all groups, with lesions observed largely in the lower lung lobes, although some of the lesions may have been the result of the postmortem BAL (Figure 3, A and B). All other gross pathology was normal except for enlarged cervical and mediastinal lymph nodes in several animals across the groups. Histological analysis of the lungs of animals in the different prophylaxis and treatment groups determined a comparable degree of pulmonary pathology when inoculated with SARS-CoV-2, similar to what had been previously published (13) (Figure 3C). Lesions were mild to moderate and characterized as multifocal interstitial pneumonia frequently centered on terminal bronchioles. The pneumonia was evident by a thickening of alveolar septae by edema fluid and fibrin and small-to-moderate numbers of macrophages and fewer neutrophils. Infiltration of small numbers of pulmonary macrophages and neutrophils were noticed in alveoli. Lungs with moderate changes also had alveolar edema and fibrin with formation of hyaline membranes. There was minimal-to-moderate type II pneumocyte hyperplasia. Occasionally, bronchioles had necrosis and loss and attenuation of the epithelium with infiltrates of neutrophils, macrophages, and eosinophils. Perivascular infiltrates of small numbers of lymphocytes forming perivascular cuffs were noticed multifocally (Figure 3C). Overall, there was no apparent difference between vehicle-treated and HCQ-treated animals in either of the regimens, prophylaxis or treatment.

**Figure 4:**
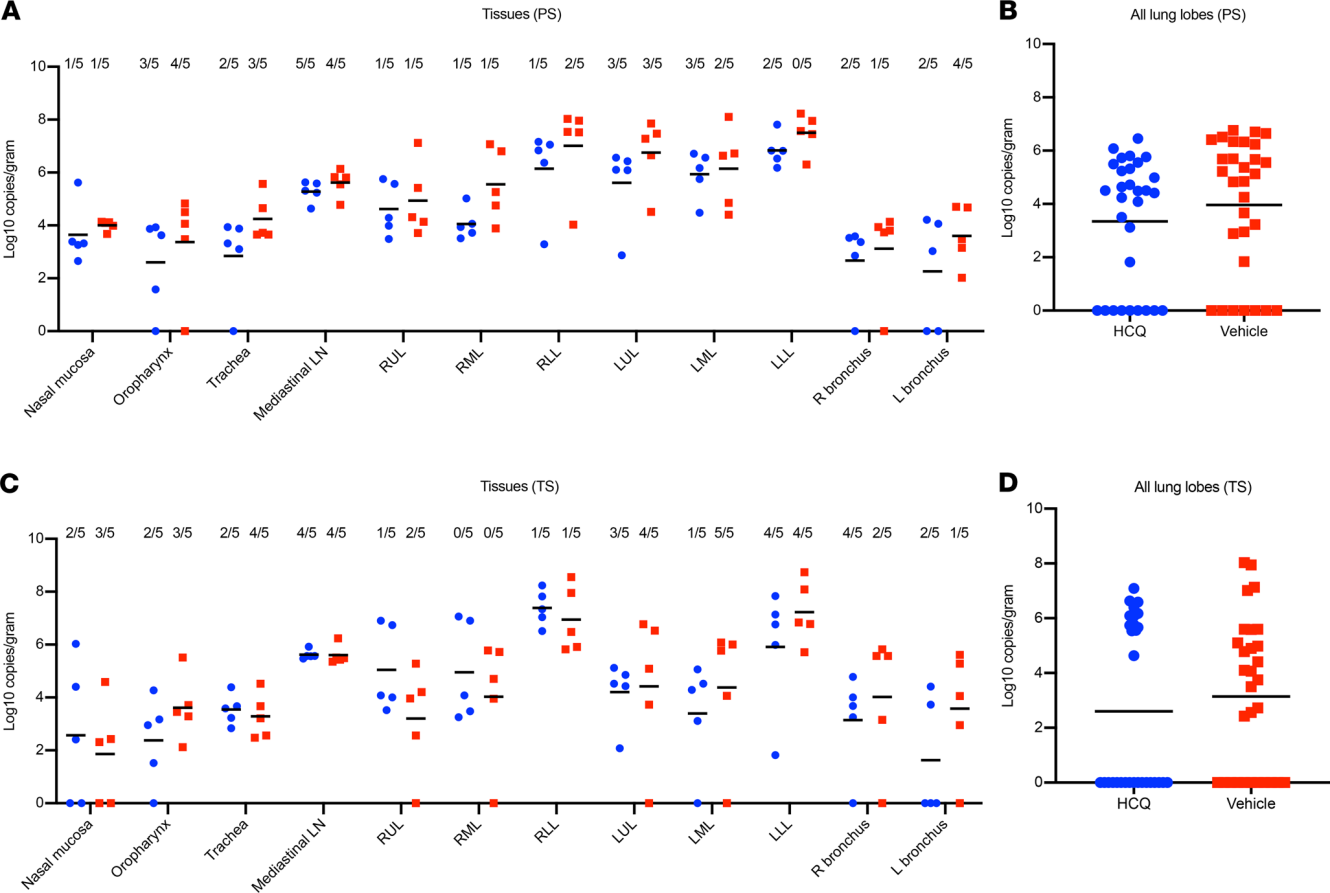
Rhesus macaque model — viral loads in respiratory tissues. Macaques were infected with SARS-CoV-2 as described in the legend of Figure 1. Animals were euthanized on day 7 after infection for viral tissue load determination performed by qRT-PCR (subgenomic copies) and virus isolation (infectious virus). (**A**) Viral loads in lower and upper respiratory tissues and mediastinal lymph nodes for the prophylaxis study arm (PS). Virus isolation is indicated in numbers on top (n/5). (**B**) Viral lung loads (PS). All lung lobe genome copy data were combined. (**C**) Viral loads in lower and upper respiratory tissues and mediastinal lymph nodes for the treatment study arm (TS). Virus isolation frequency (number of animals per group) is indicated at top (n/5). (**D**) Viral lung loads (TS). All lung lobe genome copy data were combined. No statistical significance was found among groups presented in parts **A**–**D**. A linear model was used to analyze viral RNA levels in tissues and lung lobes. No significant difference was found between groups in either study. Red squares indicate vehicle-treated animals and blue circles indicate HCQ-treated animals. SARS-CoV-2, severe acute respiratory syndrome coronavirus 2; qRT-PCR, quantitative reverse transcriptase PCR; HCQ, hydroxychloroquine; PS, prophylaxis; TS, treatment.

Viral RNA loads were determined in several respiratory tissues using qRT-PCR (Figure 4, A and C). Highest genome copy numbers were found in distal lung tissue, with a marginal but not statistically significant benefit for the HCQ-treated over the vehicle-treated group in the prophylaxis study arm when all lung lobe samples were combined (Figure 4, B and D). Virus isolation from tissues was inconsistent among animals in the different groups, but at least 1 sample in each group showed infectious virus for almost all respiratory tissues (Figure 4, A and C). There was no significant difference between vehicle-treated and HCQ-treated groups in the prophylaxis and treatment study arms, which is consistent with the lack of any observed benefit of HCQ on virus shedding parameters.

HCQ has been suggested to have immunomodulatory effects. We therefore also evaluated the lung concentrations of proinflammatory cytokines (IL-1 cytokine family, IL-2, TNF-a, IFN-g, GM-CSF, G-CSF, etc.) and CC-chemokines involved in the recruitment of mononuclear cells (MCP-1, MIP-1a, and MIP-1b) that play a role in pathogenesis and inflammatory pathology in the lung (29–31). We observed no statistically significant differences in proinflammatory cytokine or CC-chemokine concentrations among SARS-CoV-2–infected macaques either untreated or treated with HCQ (Supplemental Figures 4 and 5). Notably, IL-6 and TNF-a concentrations were undetectable in lung tissues.

**Figure 5:**
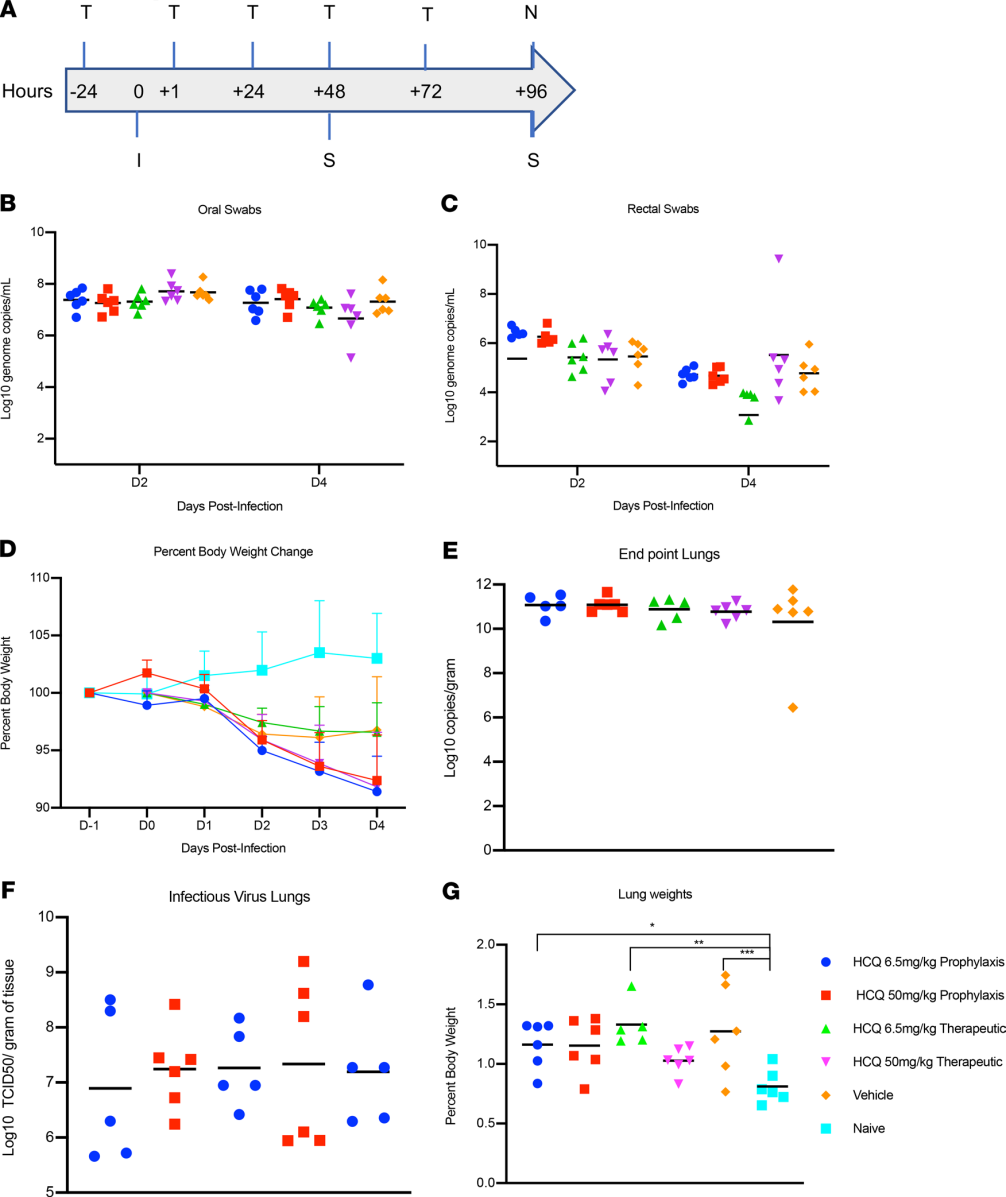
Syrian hamster model — viral shedding, viral load and pathology. Hamsters were infected with SARS-CoV-2 by the intranasal route (I). (**A**) HCQ (T) was administered either prophylactically 1 time at 24 hours before infection (6.5 mg/kg and 50 mg/kg) or as a treatment starting 1 hour after infection and continuing every 24 hours for 3 consecutive days (6.5 mg/kg and 50 mg/kg). Hamsters were scored for clinical signs daily and swabs (oral and rectal) were collected 48 and 96 hours after infection (S). Animals were euthanized on day 4 and lungs were harvested for pathology and virology. Swab and lung loads were determined by qRT-PCR. (**B** and **C**) Viral shedding. Oral and rectal swabs collected on days 2 and 4 were analyzed for viral genome copies by qRT-PCR. (**D**) Percent weight change. Hamsters were weighed daily as a parameter to measure disease progression. Daily weights were compared with starting weights to generate a percent change. (**E**) Viral load in lung tissue. Lung viral loads (E assay, genome copies) were determined as a correlate for lower respiratory tract infection. (**F**) Infectious lung titers. Lung samples were titrated for infectious virus. No statistical significance was found between the groups presented in parts **B**–**F**. (**G**) Lung-to-body weight ratio. Lung-to-body weight ratio was determined as an indicator for pneumonia with lung edema. Statistically significant differences were found only when compared with lung-to-body weight ratios of naive hamsters. SARS-CoV-2, severe acute respiratory syndrome coronavirus 2; HCQ, hydroxychloroquine; qRT-PCR, quantitative reverse transcriptase PCR.

Given this observed lack of HCQ effect in the macaque model when used either prophylactically (every 7 days) or therapeutically (daily after infection), we sought to confirm the results in a Syrian hamster SARS-CoV-2 disease model that had become available in the interim (12). In addition to the standard human dose for malaria prophylaxis/treatment (6.5 mg/kg in PBS), use of this model enabled inclusion of an additional high-dose HCQ prophylaxis/treatment regimen (50 mg/kg in PBS) to identify any dose-dependent protective activity (Figure 5A). Five groups of hamsters (*n* = 6 per group) were prophylactically or therapeutically treated with HCQ by intraperitoneal injection; control groups were treated by the same route with vehicle only. Hamsters were then intranasally infected with SARS-CoV-2 using a dose of 1 × 10^4^ TCID_50_. For prophylaxis, a single treatment was performed 24 hours before infection. The therapeutic treatment started 1 hour after SARS-CoV-2 infection and was continued for 3 consecutive days. Disease manifestation in this model is transient and clinical signs peak between days 3 and 5 after infection with ruffled fur, increased respiration rate, and reduced mobility (12). Virus replication and shedding were determined by qRT-PCR in swab samples (oral and rectal) collected on days 2 and 4, and lung tissue was taken at necropsy on day 4 after infection. Regardless of HCQ administration, all animals showed comparable high levels of genome copy numbers for oral swabs (>10^7^ genome copies/mL) and comparable lower numbers for rectal swabs (<10^6^ genome copies/mL), which decreased in all groups over time (Figure 5, B and C). Like viral RNA loads in swabs, there was no significant difference in disease manifestation over the time of the study, which can be viewed by the comparable or even greater loss in weight of the treatment groups compared with the diluent control group (Figure 5D). Gross lung pathology was similar among the groups consisting of focally extensive areas of consolidation that failed to collapse upon removal (Supplemental Figure 6). Viral lung loads on day 4 were high (10^12^ genome copies/g and >10^5^ TCID_50_/g) but indistinguishable between all groups (Figure 5, E and F). Lung-to-body weight ratios as a measure of pulmonary edema were similar in all animals, with no significant differences between groups (Figure 5G). The presence and amount of HCQ was measured in the lung tissue on day 4 after infection. Treated animals, regardless of the dosing regimen, all displayed detectable amounts of HCQ in the lung tissue. For animals for which adequate tissue mass was available for accurate measurement, HCQ levels were 2.5 ± 1.9 nmol/g and 3.5 ± 0.8 nmol/g for prophylactic low dose and high dose, respectively, and 34.3 ± 26.8 nmol/g and 107.6 ± 27.9 nmol/g for therapeutic low dose and high dose, respectively (Supplemental Table 2). Higher secondary HCQ metabolite levels relative to HCQ levels were observed in the hamster model compared with the macaque model, suggesting a more rapid drug metabolism. Despite these differences in metabolism, HCQ was present in hamster lungs at levels similar to the macaque model, supporting a similar recruitment from the bloodstream to the tissue. To estimate concentration within the tissue, a homogenous distribution was assumed with a tissue water content of 80% by weight (28). Under these constraints, the minimum calculated value equates to 1.8 μM HCQ, which falls within but at the lower end of the therapeutic window, whereas the highest calculated concentration equates to 177 μM, which is 10-fold higher than the highest reported IC_50_ (7). In summary, HCQ administered either prophylactically or as a treatment at standard or high doses reached concentrations in the infected lung tissue that were within the target range, but it did not have any significant impact on SARS-CoV-2 replication and shedding or disease manifestation and progression in the Syrian hamster model.

## Discussion

In this study, we used 2 established COVID-19–like animal models that are consistent with mild-to-moderate disease in humans (12, 13) and applied the standard weight-based oral administration of HCQ prophylaxis and treatment of malaria in humans (17, 18). The use of the Syrian hamster model also enabled inclusion of a high-dose HCQ regimen (7.5 times the standard dose regimen) both prophylactically and as a treatment to assess any dosage effect. This dose of 50 mg/kg had been previously shown to be associated with no adverse effects in Syrian hamsters (32); the lack of this assurance for rhesus macaques prevented use of such a high-dosing regimen because of veterinary concerns of toxicity. For prophylaxis, we used a weekly dosing regimen that has been standardly adopted for prevention of malaria; it is also the dosing schedule adopted by developing countries such as India with an aim toward prevention of COVID-19 (16). For treatment, we administered HCQ starting shortly after infection and continued daily until study end. HCQ pharmacokinetic studies in humans and animal models have demonstrated a rapid blood bioavailability after oral administration, with peak levels being reached in 2 to 4 hours followed by rapid absorption in various tissues including the lung (21, 26). To assess whether changes in drug metabolism after infection played a role in bioavailability, plasma samples for pharmacokinetic analysis were collected when the drug levels were at their lowest, just before the administration of the next treatment. These levels were consistent with those expected from previous animal and human studies at 12 to 24 hours after administration (21). Based on these previous models, the maximum blood concentration is likely 2 to 3 orders of magnitude higher than these minimum values. At the site of infection within the lung, the measurements taken during both studies indicate accumulation of drug at or above estimated therapeutic levels based on EC_50_ values against SARS-CoV-2 from in vitro studies (6–8, 14).

The use of HCQ and chloroquine as treatment options for patients with COVID-19 may have been partially rooted in early observations for their effect in impairing SARS-CoV-2 replication in vitro (6–8). These in vitro studies, which we confirmed herein, identified HCQ (and other 4-aminoquinolines) as potent inhibitors of coronaviruses, including SARS-CoV-2, with low EC_50_ values within the range of antivirals such as remdesivir (6), a drug that is now approved by the FDA as an Emergency Use Authorization for COVID-19 cases. The mechanism of action of 4-aminoquinolones against SARS-CoV-2 in vitro is not well defined, but increasing endosomal pH, inhibition of autophagosome-lysosome fusion, impairment of enzymes important for virus replication, and effects on protein glycosylation have been proposed, which may result in interference with SARS-CoV-2 entry/fusion, replication, and spread (33, 34). However, despite the promising in vitro effect observed by us and others, we did not observe any significant prophylactic or therapeutic benefit of HCQ after in vivo infection in 2 animal disease models. Recent studies showing cell type–specific differences in SARS-CoV-2 replication offer one possible explanation for the disconnect between in vitro and in vivo studies. Specifically, these studies have shown that the SARS-CoV-2 uses a distinct entry pathway in the Vero cells compared with the pathway used in lung epithelium in vitro and presumably in vivo. Notably, only the entry pathway in Vero cells, but not lung epithelial cells, is susceptible to in vitro inhibition by endosomal pathway inhibitors such as HCQ (35, 36).

The use of HCQ to treat COVID-19 has been controversial since the results of the first clinical trials (9, 10). Nevertheless, HCQ has been promoted as a COVID-19 treatment option and became part of multiple, recent, large-scale clinical trials, including 1 of 4 initial treatment options in the multinational WHO “Solidarity” clinical trial for COVID-19 (37). However, HCQ treatment does not come without risks; the 4-aminoquinolones are associated with multiple adverse effects such cutaneous adverse reactions, hepatic failure, and ventricular arrhythmia; overdose is also difficult to treat (17). The FDA recently updated its guidance by warning against use of HCQ outside of the hospital setting because of these potential serious adverse effects (38). HCQ treatment was recently removed from the WHO Solidarity COVID-19 clinical study based on evidence from a report, among others, from the UK-based RECOVERY trial, wherein HCQ showed no effect on the mortality rate of patients with COVID-19 (37, 39, 40). Similarly, a postexposure prophylaxis trial showed no effect of HCQ on the incidence of infection from high-risk and moderate-risk exposure to SARS-CoV-2 (15). Nevertheless, multiple preexposure prophylaxis trials remain ongoing (4). In addition, a preclinical study in cynomolgus macaques has been recently published (41). This study used daily administration of high-dose HCQ and therefore does not relate to the dosing levels used in our study or ongoing human prophylactic studies, especially in developing nations. Interestingly, in this cynomolgus macaque study, HCQ treatment did not show any significant effect on SARS-CoV-2 replication and HCQ prophylaxis did not confer protection against SARS-CoV-2 infection, which further supports our preclinical data. Clearly, the effectiveness of HCQ to prevent or reduce infection, thereby impacting the clinical course of COVID-19, remains highly contentious at this time.

In conclusion, when used at the standard dosing regimen for malaria prophylaxis and treatment, HCQ had no beneficial effect on SARS-CoV-2 replication and shedding or on disease progression and outcome in the 2 animal models tested. Potential benefits of HCQ on immunomodulation was difficult to measure in a clinically relevant fashion in our studies, as neither the macaque model nor the hamster model mimicked the presumed immune-mediated pathogenic responses seen in severe, late-stage COVID-19 cases. However, no differences were observed in any of the multiple immunomodulatory molecules tested in HCQ-treated versus nontreated macaques when measured at necropsy (day 7). There is always the consideration as to what extent animal data can be extended to the situation in humans, but in general the nonhuman primate models are considered good indicators and the ultimate preclinical models before moving drugs into clinical trials. The preclinical data presented here may help guide future decisions regarding HCQ and likely other 4-aminoquinolines in terms of the potential utility of these drugs for prophylaxis or treatment of SARS-CoV-2 infections.

## Methods

### Virus and cells.

SARS-CoV-2 isolate nCoV-WA1-2020 (MN985325.1) was provided by CDC and propagated once at Rocky Mountain Laboratories (RML) in Vero E6 cells in DMEM (MilliporeSigma) supplemented with 2% FBS (Gibco), 1 mM L-glutamine (Gibco), 50 U/mL penicillin, and 50 μg/mL streptomycin (Gibco). The used virus stock was Vero passage 4, which is free of contaminations and confirmed to be identical to the initial deposited GenBank sequence (MN985325.1). Vero E6 cells were maintained in DMEM supplemented with 10% fetal calf serum, 1 mM L-glutamine, 50 U/mL penicillin, and 50 μg/mL streptomycin.

### Rhesus macaque study design.

The study consisted of 2 arms, prophylaxis and treatment (Figure 1, A and B). Animals were anesthetized for all procedures. For the prophylaxis arm, 10 healthy rhesus macaques between 3 and 4 years of age (all male; 4.9–5.6 kg in weight) were randomly divided into vehicle control (*n* = 5) and HCQ prophylaxis (*n* = 5) groups. Animals were treated with either vehicle (PBS) or HCQ (6.5 mg/kg in PBS) 3 times 1 week apart (days –9, day –2, and day 5) by oral gavage. In the second part, 10 healthy rhesus macaques between 3 and 4 years of age (all male; 5.7–7.3 kg in weight) were randomly divided into vehicle control (*n* = 5) and HCQ treatment (*n* = 5) group. Animals were treated with either vehicle (PBS) or HCQ (6.5 mg/kg in PBS) starting 12 hours after infection followed by treatment 18, 36, 60, 84, 108, 132, and 156 hours after infection by oral gavage. All animals were infected on day 0 with a total dose of 2.8 × 10^6^ TCID_50_ of SARS-CoV-2 by a combination of 4 routes (intratracheal, oral, intranasal and ocular). Animals were monitored at least twice daily using an established scoring sheet by the same person, who was blinded to the group assignments, throughout the study (19). Physical examinations were performed on days –9, –2, 0, 1, 3, 5, and 7 and included a clinical evaluation, radiographs, venous blood draw, and swabs (oral, nasal, and rectal). BAL was performed on day 3, 5, and 7 after infection. The study endpoint was day 7. After euthanasia, necropsies were performed, and gross lung lesions were scored by a board-certified veterinary pathologist blinded to the group assignment.

### Syrian hamster study design.

The hamster study also was designed with 2 arms, prophylaxis and therapeutic. Hamsters were divided into groups for either prophylaxis treatments or therapeutic treatments (*n* = 6 per group). Two groups were treated 1 time with either a 6.5 mg/kg or 50 mg/kg 24 hours before infection for the prophylaxis arm. There were 2 therapeutic groups: 1 group received 6.5 mg/kg and the second received 50 mg/kg. Treatments began 1-hour after infection and were performed every 24 hours on days 1, 2, and 3 after infection. A final group consisted of vehicle control animals that received the same volume of PBS as the prophylactic and therapeutic groups. All groups were infected intranasally with 1 × 10^4^ TCID_50_ of SARS-CoV-2 (50 μL/nare). Animals were weighed daily and the D0 weight was used as the baseline to calculate percent weight change over the course of the study. Animals were euthanized and samples were collected on day 4 after infection. All procedures were performed on anesthetized animals. Swabs (oral, rectal) were collected on days 2 and 4, and lung tissues were collected at necropsy on day 4 after infection for pathology and virology.

### Liquid chromatography and mass spectrometry (LC–MS).

LC–MS grade water, methanol, acetonitrile, and formic acid were purchased through Fisher Scientific. All synthetic standards for molecular analysis were purchased from Santa Cruz Biotechnology. Levels of HCQ and secondary metabolites were determined using methodology established previously with modifications (23). Plasma and cleared lung homogenates were γ-irradiated (2 × 10^4^ Gy) before removal from biocontainment according to IBC-approved protocol. Plasma samples were prepared for small molecule analysis by diluting a 25 μL aliquot with 100 μL of 0.1% formic acid and 1 mL of acetonitrile on ice. Clarified lung homogenate samples were prepared by adding 1 mL of acetonitrile to a 250 μL aliquot of lung homogenate. Samples were vortexed and incubated at –20°C for 2 hours. Samples were centrifuged at 16,000*g* and 4^o^C for 20 minutes. The clarified supernatants (1 mL) were recovered and taken to dryness in a Savant DNA120 SpeedVac concentrator (Thermo Fisher Scientific). Samples were resuspended in 100 μL of 50% methanol and 50% water (v/v) and centrifuged as before. The supernatant was taken to a sample vial for LC–MS analysis. Samples were separated by reverse-phase chromatography on a Sciex ExionLC AC system. Samples were injected onto a Waters Atlantis T3 column (100Å, 3 μm, 3 mm × 100 mm) and eluted using a binary gradient from 25% methanol, 0.1% formic acid to 100% methanol formic acid over 4 minutes. Analytes were measured using a Sciex 5500 QTRAP mass spectrometer in positive mode. Multiple reaction monitoring was performed using previously established signal pairs for each analyte and signal fidelity was confirmed by collecting triggered product ion spectra and comparing back to spectra of synthetically pure standards. All analytes were quantified against an 8-point calibration curve of the respective synthetic standard prepared in the target matrix and processed in the same manner as experimental samples. Limits of quantification in plasma for all metabolites was 0.5 ng/mL. Limit of quantification in lung homogenate for all metabolites was 6 ng/mL apparent and sample data were filtered before weight normalization.

### Hematology and serum chemistry.

Hematology was completed on a Procyte DX (IDEXX Laboratories) and the following parameters were evaluated: RBCs, hemoglobin, hematocrit, mean corpuscular volume, mean corpuscular hemoglobin (MCH), MCH concentration, red cell distribution weight, platelets, mean platelet volume, WBCs, neutrophil count (abs and %), lymphocyte count (abs and %), monocyte count (abs and %), eosinophil count (abs and %), and basophil count (abs and %). Serum chemistries were completed on a Vetscan VS2 Chemistry Analyzer (Abaxis) and the following parameters were evaluated: glucose, blood urea nitrogen, creatinine, calcium, albumin, total protein, alanine aminotransferase, aspartate aminotransferase, alkaline phosphatase, total bilirubin, globulin, sodium, potassium, chloride, and total carbon dioxide.

### Thoracic radiographs.

Ventro-dorsal and right/left lateral radiographs were taken of nonhuman primates on clinical exam days and scored for the presence of pulmonary infiltrates by 2 clinical veterinarians according to a standard scoring system (0, normal; 1, mild interstitial pulmonary infiltrates; 2, moderate pulmonary infiltrates perhaps with partial cardiac border effacement and small areas of pulmonary consolidation; 3, serious interstitial infiltrates, alveolar patterns, and air bronchograms). Individual lobes were scored. Scores from the lobes were then totaled and recorded per animal per day.

### Virus load.

RNA was extracted from swabs and BAL using the QIAamp Viral RNA Kit (QIAGEN) according to the manufacturer’s instructions. Tissues were homogenized in RLT buffer and RNA was extracted using the RNeasy Kit (QIAGEN) according to the manufacturer’s instructions. For detection of viral RNA, 5 μl RNA was used in a 1-step real-time RT-PCR assay for total RNA using E (13) or an E subgenomic mRNA (42) using the Rotor-Gene Probe Kit (QIAGEN) according to instructions of the manufacturer. In each run, standard dilutions of RNA standards counted by droplet digital PCR were run in parallel, to calculate copy numbers in the samples.

### Virus titration.

Virus isolation was performed on lung tissues by homogenizing the tissue in 1 mL DMEM using a TissueLyser (QIAGEN) and inoculating Vero E6 cells in a 24-well plate with 250 μL of cleared and a 1:10 dilution of the homogenate. One hour after inoculation of cells, the inoculum was removed and replaced with 500 μL DMEM (MilliporeSigma) supplemented with 2% FBS, 1 mM L-glutamine, 50 U/mL penicillin, and 50 μg/mL streptomycin. Six days after inoculation, cytopathogenic effect was scored and the TCID_50_ was calculated.

### Cytokine analysis.

A small lung sample was excised from every lung lobe from each macaque and placed into 1mL plain DMEM (6 samples per macaque). To disrupt cell membranes, tissue samples were sonicated and then centrifuged to clarify the supernatant. Supernatants were then collected and stored at −80 °C until use. Lung homogenates were diluted at 1/4 with plain DMEM. The concentrations of cytokines and chemokines present in the lung homogenates were quantified using the MILLIPLEX MAP Non-Human Primate Cytokine Magnetic Bead Panel - PCYTMG-40 K- Cytokine-Chemokine Array Kit (Millipore) following the manufacturer’s instructions. The analytes detected by this panel are as follows: G-CSF, GM-CSF, IFN-g, IL-1ra, IL-1b, IL-2, IL-4, IL-5, IL-6, IL-8, IL-10, IL-12/23 (p40), IL-13, IL-15, IL-17, IL-18, MCP-1, MIP-1a, MIP-1b, sCD40L, TGF-a, TNF-a, and VEGF. The multiplex plate was read using a Bio-Plex 200 Suspension Array Luminex System (Bio-Rad). Data were normalized based on the tissue weight.

### Histopathology and IHC.

Histopathology and IHC were performed on rhesus macaque tissues. After fixation for a minimum of 7 days in 10% neutral-buffered formalin and embedding in paraffin, tissue sections were stained with H&E. Tissues were placed in cassettes and processed with a Sakura VIP-6 Tissue Tek on a 12-hour automated schedule, using a graded series of ethanol, xylene, and ParaPlast Extra. Embedded tissues were sectioned at 5 μm and dried overnight at 42°C before staining. Specific anti-CoV immunoreactivity was detected using GenScript U864YFA140-4/CB2093 NP-1 at a 1:1000 dilution. The secondary antibody was an anti–rabbit IgG polymer from Vector Laboratories ImPress VR. Tissues were then processed for IHC using the Discovery Ultra automated processor (Ventana Medical Systems) with a ChromoMap DAB Kit (Roche Tissue Diagnostics). Stained slides were analyzed by a board-certified veterinary pathologist.

### Statistics.

Statistical analysis was performed in Prism 8 (GraphPad). Two-tailed *t* tests were used to compare experiments consisting of 2 experimental groups. One-way ANOVA was used to analyze all data sets with more than 2 experimental groups. A *P* value of less than 0.05 was considered significant.

### Study approval.

Work with infectious SARS-CoV-2 was approved by the Institutional Biosafety Committee (IBC) and performed in high biocontainment at RML, National Institute of Allergy and Infectious Diseases (NIAID), NIH. Sample removal from high biocontainment followed IBC-approved standard operating protocols. Animal work was approved by the Institutional Animal Care and Use Committee and performed by certified staff in an AALAC International-accredited facility. Work followed the institution’s guidelines for animal use, the guidelines and basic principles in the NIH *Guide for the Care and Use of Laboratory Animals* (National Academies Press, 2011), the Animal Welfare Act, US Department of Agriculture, and the US Public Health Service Policy on Humane Care and Use of Laboratory Animals. Syrian hamsters were group-housed in HEPA-filtered cage systems enriched with nesting material and were provided with commercial chow and water ad libitum. Nonhuman primates were single-housed in adjacent primate cages, allowing social interactions, in a climate-controlled room with a fixed light/dark cycle (12-hour light/12-hour dark). They were provided with commercial monkey chow, treats, and fruit twice daily with water ad libitum. Environmental enrichment consisted of a variety of human interaction, manipulanda, commercial toys, videos, and music. Hamsters and nonhuman primates were monitored at least twice daily.

## Author contributions

KR, MAJ, FF, and HF contributed to the design, execution of each study, data analysis, and preparation of the manuscript. BS and KAS contributed to the metabolite analysis and editing. JL and PWH contributed to the veterinary care, animal exams, and administration of treatments in the NHP study. GS and DS contributed to the pathological analysis of each study. AO, KMW, BNW, FH, LPP, LS, TLTH, JC, and EH contributed experiment support and data analysis. GS, CMB, DH, and EDW all contributed to data analysis and/or experimental design.

## Supplementary Material

Supplemental data

## Version 1 10/22/2020

In-Press Preview

## Version 2 12/03/2020

Electronic publication
